# Disorders Related to PI3Kδ Hyperactivation: Characterizing the Clinical and Immunological Features of Activated PI3-Kinase Delta Syndromes

**DOI:** 10.3389/fped.2021.702872

**Published:** 2021-08-05

**Authors:** Vyanka Redenbaugh, Tanya Coulter

**Affiliations:** ^1^Regional Immunology Services of Northern Ireland, Belfast Health and Social Care Trust, Belfast, United Kingdom; ^2^Mayo Clinic, Rochester, MN, United States

**Keywords:** *PIK3CD*, *PIK3R1*, phosphatase and tensin homolog, activated PI3K delta syndrome, PI3 K

## Abstract

Phosphoinositide-3-kinase δ (PI3Kδ) is found in immune cells and is part of the PI3K/AKT/mTOR/S6K signalling pathway essential to cell survival, growth and differentiation. Hyperactivation of PI3Kδ enzyme results in Activated PI3-kinase delta syndrome (APDS). This childhood onset, autosomal dominant, combined immunodeficiency, is caused by heterozygous gain of function (GOF) mutations in *PIK3CD* (encodes PI3Kδ catalytic subunit p110δ), mutations in *PIK3R1* (encodes PI3Kδ regulatory subunit p85α) or LOF mutations in *PTEN* (terminates PI3Kδ signalling) leading to APDS1, APDS2 and APDS-Like (APDS-L), respectively. APDS was initially described in 2013 and over 285 cases have now been reported. Prompt diagnosis of APDS is beneficial as targeted pharmacological therapies such as sirolimus and potentially PI3Kδ inhibitors can be administered. In this review, we provide an update on the clinical and laboratory features of this primary immunodeficiency. We discuss the common manifestations such as sinopulmonary infections, bronchiectasis, lymphoproliferation, susceptibility to herpesvirus, malignancy, as well as more rare non-immune features such as short stature and neurodevelopmental abnormalities. Laboratory characteristics, such as antibody deficiency and B cell and T cell, phenotypes are also summarised.

## Key Points/Practical Pearls

- APDS is a childhood onset, clinically heterogenous disorder.
- Patients may initially be diagnosed with Hyper IgM syndrome, CVID, combined immunodeficiency, specific antibody deficiency or autoimmune lymphoproliferative syndrome-like disorder.
- The immunodeficiency phenotype in APDS can be predominantly antibody deficiency, with recurrent sinopulmonary tract infections, or combined immunodeficiency, with a predisposition to herpesvirus in addition to bacterial infections.
- Benign lymphoproliferation can manifest as lymphoid hyperplasia, lymphadenopathy, splenomegaly or hepatomegaly.
- The most common autoimmune manifestation is autoimmune cytopenia (ITP or AIHA).
- B cell lymphomas are the most common malignancy and are often associated with EBV infection.
- Clinical manifestations of APDS typically progress from recurrent infections and lymphoproliferation in early childhood, to autoimmunity in mid-childhood, and malignancy in late childhood-adulthood.
- Short stature and neurodevelopmental delay are more common in APDS2 compared with APDS1.
- Hyper IgM is the most common immunoglobulin pattern, but a range of antibody defects can occur; hypogammaglobulinaemia, agammaglobulinaemia, IgG subclass deficiency, specific antibody deficiency (poor response to pneumococcal polysaccharide vaccination), and normal immunoglobulin levels. Therefore, a diagnosis of APDS should not be ruled out based on immunoglobulin levels alone.
- Lymphocyte subset testing typically reveals B cell lymphopenia, CD4+ T cell reduction, with an inverted CD4/CD8 ratio.
- Diagnosis is made by detecting a heterozygous mutation in *PIK3CD* (APDS1), *PIK3R1* (APDS2) or *PTEN* (APDS-L).
- Treatment is tailored according to the clinical phenotype and includes prophylactic antibiotics, immunoglobulin replacement therapy, immunosuppression (steroids, rituximab, sirolimus) and HSCT. Selective PI3Kδ inhibitors such as leniolisib are emerging treatments.
- Early recognition and disease diagnosis are important in trying to prevent long term complications such as bronchiectasis, hearing loss, and malignancy.
- Fatalities can occur as a result of infection or malignancy.
- Patients should have regular monitoring to detect cytopenias, bronchiectasis, and malignancy.
- Patients should be managed by immunology services in conjunction with respiratory services, particularly if bronchiectasis is present. Input from other specialists, such as haematology, infectious diseases and gastroenterology, may be required. Children with APDS should also have a general paediatrician.
- PTEN deficiency causes PHTS and can also manifest as an APDS-like disorder. APDS-L patients have a phenotype similar to APDS with lymphoproliferation, autoimmunity, and malignancy. However, they have an increased frequency of autoimmune thyroiditis and solid organ tumours, in contrast to cytopenias and B cell lymphomas.

## Background

In this review, we discuss the clinical and immunological features of Activated PI3-Kinase Delta Syndromes - Activated PI3-Kinase Delta Syndrome 1 (APDS1) and Activated PI3-Kinase Delta Syndromes 2 (APDS2) and the APDS-Like (APDS-L) condition PTEN deficiency.

**Figure 1:**
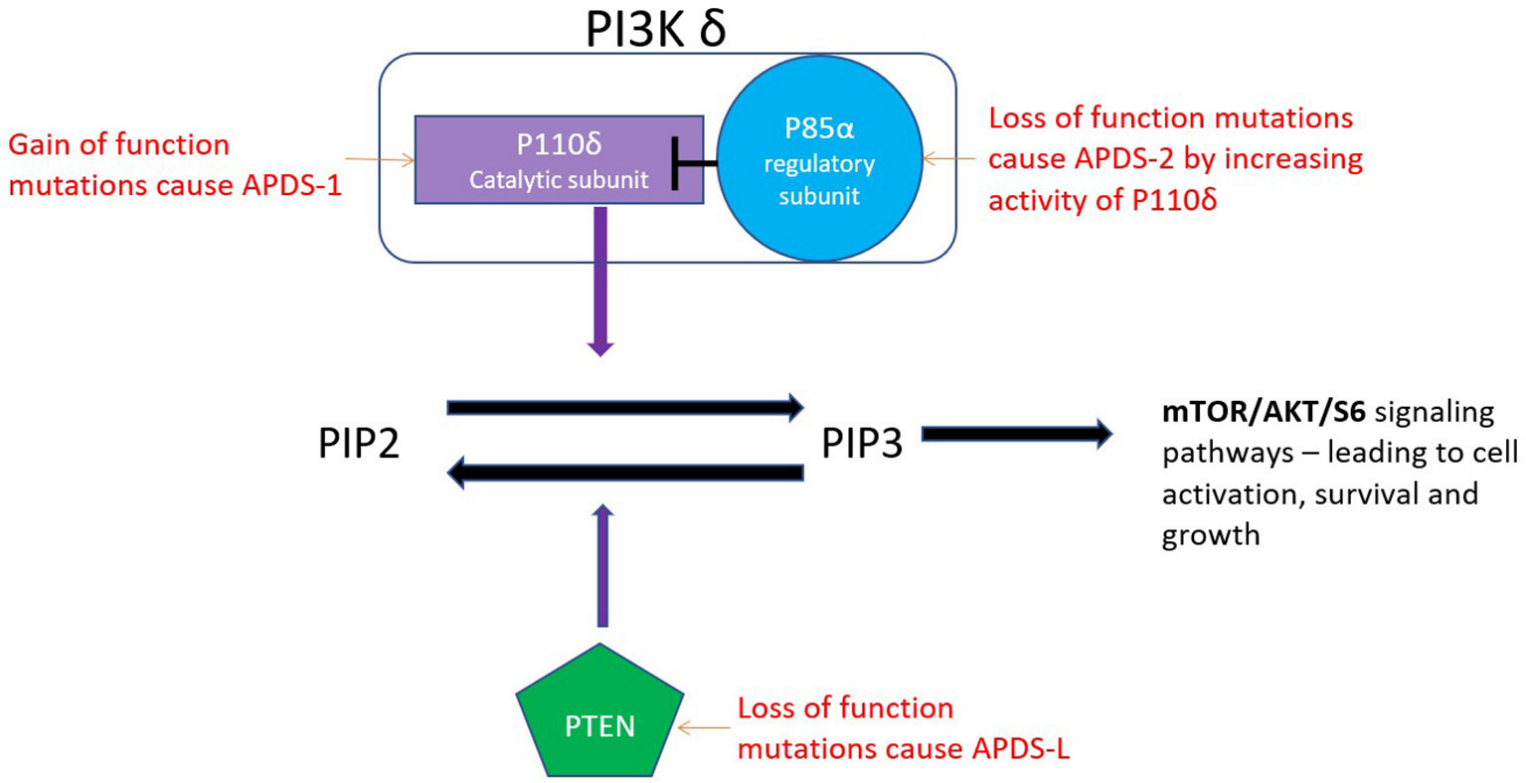
The catalytic subunit (P110 δ) of the PI3K δ enzyme converts PIP2 to PIP3. PTEN converts the PIP3 back to PIP2.

PI3-kinase δ is a class 1 phosphoinositide-3-kinase consisting of the catalytic subunit p110δ and, most commonly, the regulatory subunit p85α, although association with other regulatory subunits is also possible (1). p110δ is expressed primarily in haematopoietic cells and cells of the nervous system, whereas p85α expression is more ubiquitous (1, 2). PI3Kδ is activated by antigen receptors, co-receptors, growth receptors and cytokine receptors. Activation catalyses the phosphorylation of phosphatidylinositol 3,4-bisphosphate (PIP2) to generate phosphatidylinositol 3,4,5-triphosphate (PIP3) (Figure 1) (1). This leads to cell activation, growth, metabolism and inhibition of apoptosis via the AKT/mTOR/S6K signalling pathways (2).

**APDS1** is caused by autosomal dominant, gain of function (GOF) mutations in the p110δ catalytic subunit (encoded by *PIK3CD*). The most frequently reported mutation in APDS1 is the p.E1021K missense mutation (3). APDS 1 mutations have high penetrance with only 1 of 53 patients in the largest described cohort being asymptomatic (3). PI3Kδ is dynamically regulated and demonstrates the fine balance of the immune system as underactivation, as well as overactivation, leads to immunodeficiency. Germline biallelic loss of function (LOF) mutations leading to underactivation of PI3Kδ present with infections, colitis, panhypogammaglobulinemia and lymphopenia (1).

**APDS2** is caused by autosomal dominant mutations in *PIK3R1* which encodes the PI3Kδ regulatory subunit p85α. Mutant p85α is less able to inhibit PI3Kδ as wild-type p85α (4). This allows for hyperactivation of the catalytic subunit P110δ. Therefore, LOF mutations of the regulatory subunit result in overall gain of function of the PI3Kδ enzyme. Over 285 cases of APDS1 and 2 have been described in the literature.

**APDS-L** is caused by heterozygous LOF mutations in phosphatase and tensin homologue (PTEN). PTEN is a lipid phosphatase that converts PIP3 back to PIP2, hence terminating the signal initiated by PI3Kδ activation. PTEN was initially described as a tumour suppressor gene (5). Autosomal dominant LOF mutations in PTEN (encoded by *PTEN*) lead to PTEN hamartoma tumour syndrome (PHTS) which encompasses a variety of syndromes predisposing to cancer, such as Cowden syndrome and Bannayan–Riley–Ruvalcaba syndrome (6). A study of 79 individuals with PHTS did not report an increased rate of infections in this condition (7). It was only after the description of APDS that a causative link between LOF PTEN mutations and immunodeficiency was explored and individuals with recurrent infections and PTEN deficiency were discovered (8–10). This phenotype is referred to as APDS-L (6). In individuals with APDS-L, PTEN expression in activated T cells is reduced (~60% of normal) and lower degrees of AKT hyperactivation have been shown, compared with APDS1 and APDS2 (7, 11, 12). This is perhaps why immunodeficiency was rarely previously described in patients with PHTS (7). Homozygous loss of function of PTEN is incompatible with life (13).

APDS 1, APDS2 and PTEN deficiency (APDS-L) are classified as predominantly antibody deficiencies by the International Union of Immunological Societies Expert Committee (14) as the most common manifestation is bacterial infections of the respiratory tract. However, in some individuals the susceptibility to severe viral infection suggests APDS can also be phenotypically similar to a combined immunodeficiency.

### Age of Onset and Progression of Symptoms

APDS is a childhood onset immunodeficiency. Jamee et al. (15) on systematic review reported a median age of onset of 1.6 years [Inter-quartile range (IQR) 0.58–3.0 yrs] with significant delay in diagnosis of 7.0 years (IQR 3.4–14.0yrs). Jamee et al. also described that the various clinical manifestations of APDS typically present at different ages with recurrent infections beginning in the first year of life (median age 1.16 yrs) and lymphoproliferation developing in early childhood (median age 3 yrs). Autoimmunity presents in later childhood, while malignancy can occur at any age (3, 15, 16) but most commonly in late childhood - early adulthood (median age 18 yrs).

### Clinical Features

#### Respiratory Tract Infections

Recurrent sinopulmonary tract infections are the most common clinical manifestation of APDS, affecting almost all patients described in the largest studies (3, 16–22) (see Table 1 for clinical features).

**Table 1:** Clinical features of APDS1, APDS2 and APDS-L.

	**APDS 1**	**APDS 2**	**APDS –L (PTEN deficiency)**
**Infections**	✓✓✓	✓✓✓	✓
Bacterial sinopulmonary	✓✓	✓✓	
Herpesvirus			
**Benign lymphoproliferation**	✓✓✓	✓✓✓	✓✓✓
**Autoimmunity**	✓✓	✓✓	✓
Cytopenia	✓	✓	✓✓
Other^*^			
**Malignancy**	✓✓	✓✓	✓✓
Lymphoma			
Other^†^			
**Short stature**	✓	✓✓	
**Neurodevelopmental delay**	✓	✓✓	✓✓✓

* *Autoimmune thyroiditis, glomerulonephritis, diabetes, pancreatitis, hepatitis, colitis, arthritis*.

† *Basal cell carcinoma, dysgerminoma, rhabdomycosarcoma, melanoma, carcinoma of breast, endometrium, thyroid, kidney, colorectum*.

Respiratory tract infections include pneumonia (43%), sinusitis (29%) and otitis media (26%) (15).

Other upper respiratory tract complications, including adenoid and tonsillar hypertrophy and parotitis also occur (3, 15). Recurrent tonsillitis often results in tonsillectomy (3). In some cases, otitis media is severe enough to cause permanent hearing loss (3, 20).

Around 50% of patients with APDS develop bronchiectasis on a background of recurrent respiratory tract infections (3, 19).

Small airway damage and mosaic attenuation on CT is also commonly described and thought to be the consequence of repeated episodes of viral bronchiolitis compounded by local obstruction secondary to focal lymphoid hyperplasia (3, 23).

APDS-L (PTEN deficiency) has presented with recurrent upper and lower respiratory tract infections and pan-hypogammaglobulinemia (8–10).

#### Bacterial Infections

Significant bacterial infections at a variety of sites have been reported in APDS.

There is a predisposition to eye infections with reports of a variety of ocular infections including orbital cellulitis (3, 16, 18).

Septicaemia, meningitis, infectious lymphadenitis, osteomyelitis, dental abscesses and Staphylococcus aureus skin abscesses are also described (15, 16, 19, 24).

The most commonly cultured bacteria are those typical in antibody deficiencies, *Streptococcus pneumoniae, Haemophilus influenzae* and *Staphylococcus aureus* (3, 15).

In a Chinese cohort, Wang et al. found 20% (3 of 15) patients had tuberculous infections prior to diagnosis of APDS1 (20). Persistent local granulomatosis skin reactions to BCG vaccination have been described in 3 of 10 known BGC vaccinated patients with APDS1, and 2 of 18 BCG vaccinated patients with APDS2 (3, 18, 23, 25). Recently, Fekrvand et al. (2021) reported a patient with APDS2 who succumbed to a fatal BCG infection following BCG vaccination (22).

#### Viral Infections

##### Herpesvirus

The PI3K pathway has a critical role in herpesvirus infection, and the control of herpesviruses by the immune system. Herpesviruses manipulate this pathway to enhance virus entry, replication, latency, and reactivation (26). In APDS the most frequent infections are due to Epstein-Barr virus (EBV), presenting as persistent EBV viremia or EBV lymphoproliferation (26). Cytomegalovirus (CMV) infection can also be common, presenting as persistent CMV viremia or lymphadenitis (26). Sporadic cases of severe herpes simplex viral infections, including pneumonia and keratitis, have been reported (3). An increased frequency of herpesvirus infections has not been reported in APDS-L (6–10).

##### Other Viral Infections

Extensive warts caused by papillomavirus and molluscum contagiosum lesions have been reported (3, 16, 18, 27). Adenovirus and norovirus infections have also been described (3).

#### Fungal Infections

Patients with APDS can develop mucocutaneous candidiasis (3.5% in Jamee et al.) (3, 15, 18).

An increased frequency of fungal infections has not been reported in APDS-L (7–10).

#### Lymphoproliferation

Benign lymphoproliferation is the second most common manifestation of APDS (after respiratory tract infections), present in the majority of patients (59–93%) (3, 15, 16, 19, 20, 24). Lymphadenopathy occurs most frequently (61%), followed by splenomegaly (47%) and hepatomegaly (29%) (15). Lymphadenopathy can be persistent or recurrent, and is often localised to sites of infection (3).

Reactive hyperplasia and lymphadenitis can occur in the gastrointestinal and respiratory tracts in APDS (32% of patients, Coulter et al.) (3). Tonsillar and adenoid hypertrophy can require multiple surgical excisions (18).

Interestingly, lymphoid hyperplasia has been reported in 24–47% of patients with APDS- L (7, 28). This is usually hyperplasia of the adenoids, tonsils and gastrointestinal tract, with lymphadenopathy also described (7, 22, 28, 29).

#### Autoimmunity

Autoimmunity is present in just under one-third of patients (15). **Cytopenias** are the most common autoimmune manifestation (accounting for 76% of all autoimmune complications) and include immune thrombocytopenia purpura (ITP) and autoimmune haemolytic anaemia (AIHA) (3, 15, 19).

Autoimmune conditions affecting various other organs have been reported, including autoimmune thyroiditis, glomerulonephritis, nephrotic syndrome, insulin-dependent diabetes, exocrine pancreatic insufficiency, autoimmune hepatitis, arthritis, Sjogren's syndrome and pericarditis (3, 18, 19, 21).

Autoantibodies have been reported in 4 of 23 (17%) and 3 of 53 (5%) in two APDS1 series (3, 21).

APDS-L has a similar frequency of autoimmunity (27–32% of APDS-L cases) to APDS (7, 28). However, in APDS-L, thyroiditis is much more common than autoimmune cytopenia, accounting for 36–81% and 5–9% of autoimmune manifestations in APDS-L, respectively (7, 28).

#### Malignancy

The most common malignancies reported in APDS are B cell lymphomas; diffuse large B cell lymphoma (DLBCL), Hodgkin lymphoma and marginal zone B cell lymphoma all being described. The frequency of lymphoma appears higher in patients with a history of chronic viral infections, with chronic EBV reported in almost half of the patients who developed lymphoma (15).

Non-haematological malignancies have also been reported; basal cell carcinoma, dysgerminoma, rhabdomyosarcoma, and papillary neoplasm of the breast (18, 19, 30, 31).

As *PTEN* is a tumour suppressor gene, PTEN deficiency increases the risk of malignancy independently of an APDS-L phenotype (6). Patients are at increased risk of breast, thyroid, kidney, endometrial, colorectal cancer, and melanoma with an estimated lifetime risk of 85, 35, 33, 28, 9, and 6%, respectively (12), whereas B cell lymphomas are less commonly described.

#### Gastrointestinal and Hepatic Features

Enteropathy occurs in patients with APDS in ~25% of cases, manifesting as diarrhoea and / or malabsorption (3, 15). The underlying pathology can be autoimmune, infectious or both.

Ben-Yakov et al. observed transaminitis in 9 out of 33 (27%) APDS patients (32). Four of five patients had liver biopsies on which features were suggestive of nodular regenerative hyperplasia (NRH) and mildly increased portal pressure (32). Primary sclerosing cholangitis has also been reported in APDS (3, 33).

In a series of 34 patients with PTEN mutations (APDS-L), gastrointestinal lymphoid hyperplasia (*n* = 16) and indeterminate colitis (*n* = 1) were described (28). Chen et al. also found colitis in 5% of the 79 patients with PTEN mutations described in their case series (7).

#### Neurodevelopmental Features

PI3Kδ is expressed broadly in the developing central nervous system of mice (34). Neurological abnormalities, neurodevelopmental delay and autism spectrum disorders, are described in APDS and are more common in APDS2 (26.6%) than APDS1 (9.5%) (15). Neurodevelopmental delay may present as mild cognitive impairment or learning disabilities (21). Macrocrania has been described in APDS1 (3, 24) and microcephaly in APDS2 (35).

PTEN deficiency is commonly associated with macrocephaly (80–100%) and developmental delay (12–20%) (36). Autism spectrum disorders are also described (29).

#### Other Non-Infectious Features

**Short stature** has been observed frequently (45–60%) in APDS2 (6, 18) but not APDS1 (4.5%) (15). This difference may be due to p85α (the effected subunit in APDS2) being more ubiquitously expressed in cells than p110δ (the effected subunit in APDS1). Mutations in *PIK3R1* that lead to underactivation of PI3K are known to cause SHORT syndrome (short stature, hyperextensibility of joints, ocular depression, Rieger anomaly, and teething delay syndrome) (37).

Eczema was described in 20% of patients with APDS in the ESID-APDS registry (19).

### Immunological Characteristics of APDS1 and APDS2

#### Immunoglobulins

APDS can present with a variety of different immunoglobulin profiles. IgG and IgA levels are commonly low or normal, while IgM can be high or normal. A hyper IgM-like pattern is the most common immunoglobulin profile reported (low IgG, low IgA and high IgM). This is explained by the fact that PI3Kδ signalling pathways are involved in immunoglobulin class switching (25) with class switched memory B cells reduced in APDS. Hypogammaglobulinaemia, agammaglobulinaemia, selective IgA deficiency and specific antibody deficiency are the other immunoglobulin patterns found in APDS (3, 25).

Patients can also have normal immunoglobulin levels, therefore, the diagnosis of APDS cannot be ruled out based on immunoglobulin profile alone.

#### Vaccine Responses

Vaccine responses are often impaired (3, 16). Defective anti-polysaccharide vaccine responses were detected in 52 of 58 patients (90%) and defective anti-peptide antibody responses in 14 of 42 (33%) (15).

#### Lymphocyte Studies

Lymphopenia occurs in around one third of patients (15). Low B cell counts are reported in the majority of patients (67–88%) (3, 15, 17, 19). T cell profiles often show a reduction in CD4+ T cells (1, 3, 17), with an inverted CD4/CD8 ratio (3, 17, 23). Mild NK cell deficiency may also be present (20–27%) (3, 20).

Extended lymphocyte phenotypes have shown: reduced naive CD4+ T cells (CD4+CD45RA+); increased activated CD8+ T cells (CD8+CD45RO+); reduced class-switched memory B cells and expanded transitional B cells (15).

Kang et al. reported impaired lymphocyte proliferation to PHA in 3/3 of their patients (24).

### Immunological Features in PTEN Deficiency (APDS-L)

Immunological abnormalities such as reduced CD4+ T cells, reduced CD4:CD8 ratio, or altered immunoglobulin levels are described and can often be detected even in patients without clinical immunodeficiency (7, 28).

## Treatment

Due to the wide clinical spectrum of APDS, treatment is personalised to the severity of the clinical phenotype. Asymptomatic family members may need no treatment at all (38). Patients who present primarily as antibody deficiencies are treated with immunoglobulin replacement therapy and/or prophylactic antibiotics.

Immunosuppressive treatments (steroids, rituximab etc.) have been used to manage autoimmune cytopenias and benign lymphoproliferation (38). Sirolimus inhibits mTOR (downstream of PI3K) and acts as a more targeted immunosuppressive agent in lymphoproliferation.

Haematopoetic stem cell transplantation (HSCT) remains the only curative treatment but does have a significant associated mortality rate (27). HSCT may be indicated in cases with severe infection or lymphoma. 12.8% of reported cases have underwent HSCT (15). A recent study by Dimitrova et al. showed that post HSCT 2-year overall and graft failure–free survival probabilities were 86 and 68%, respectively, and did not differ significantly by APDS1 vs. APDS2, donor type, or conditioning intensity. Interestingly, they found the use of rapamycin/mTOR in first year post transplant resulted in more graft failure (42 vs. 9% without mTOR) and increased incidence of unplanned donor cell infusion (65% with mTOR vs. 23% without) (39).

Selective PI3Kδ inhibitors are emerging treatments. Leniolisib is an oral small molecule inhibitor of PI3Kδ that is in phase II clinical trials. Six patients received leniolisib as part of an open label trial (40), which suggested it could be beneficial, particularly for treating lymphoproliferation (40). Leniolisib has been given orphan drug designation by the European Commission and US Food and Drug Administration.

## Author Contributions

Both authors listed have made a substantial, direct and intellectual contribution to the work, and approved it for publication.

## Conflict of Interest

The authors declare that the research was conducted in the absence of any commercial or financial relationships that could be construed as a potential conflict of interest.

## Publisher's Note

All claims expressed in this article are solely those of the authors and do not necessarily represent those of their affiliated organizations, or those of the publisher, the editors and the reviewers. Any product that may be evaluated in this article, or claim that may be made by its manufacturer, is not guaranteed or endorsed by the publisher.
